# Toxicity and quality of life after choline-PET/CT directed salvage lymph node dissection and adjuvant radiotherapy in nodal recurrent prostate cancer

**DOI:** 10.1186/1748-717X-9-178

**Published:** 2014-08-12

**Authors:** Cordula A Jilg, Anja Leifert, Daniel Schnell, Simon Kirste, Natalia Volegova-Neher, Daniel Schlager, Gesche Wieser, Karl Henne, Wolfgang Schultze-Seemann, Anca-L Grosu, Hans Christian Rischke

**Affiliations:** Department of Urology, Albert-Ludwigs University of Freiburg, Hugstetterstr. 55, 79106 Freiburg, Germany; Department of Radiation Oncology, Albert-Ludwigs University of Freiburg, Robert-Koch-Str. 3, 79106 Freiburg, Germany; Department of Nuclear Medicine, Albert-Ludwigs University of Freiburg, Hugstetterstr. 55, 79106 Freiburg, Germany

**Keywords:** Adjuvant radiotherapy, Prostate cancer relapse, Toxicity of radiotherapy, Lymph node metastases, Salvage lymphadenectomy, Choline PET, PET/CT

## Abstract

**Background:**

In a previous study we demonstrated that, based on ^11^C/^18^ F-choline positron emission tomography-computerized-tomography as a diagnostic tool, salvage lymph node dissection (LND) plus adjuvant radiotherapy (ART) is feasible for treatment of pelvic/retroperitoneal nodal recurrence of prostate cancer (PCa). However, the toxicity of this combined treatment strategy has not been systematically investigated before. The aim of the current study was to evaluate the acute and late toxicity and quality of life of ART after LND in pelvic/retroperitoneal nodal recurrent PCa.

**Material and methods:**

43 patients with nodal recurrent PCa were treated with 46 LND followed by ART (mean 49.6 Gy total dose) at the sites of nodal recurrence. Toxicity of ART was analysed by physically examination (31/43, 72.1%), by requesting 15 frequent items of adverse events from the Common-Terminology-Criteria for Adverse Events Version 4.0-catalogue and by review of medical records. QLQ-C30 (EORTC quality of life assessment) and PR25 (prostate cancer module) questionnaires were used to investigate quality of life. Toxicity was evaluated before starting of ART, during ART (acute toxicity), after ART (mean 2.3 months) and at end of follow up (mean 3.2 years after end of ART) reflecting late toxicity.

**Results:**

71.7% (33/46) of 46 ART were treatment of pelvic, 10.9% (5/46) of retroperitoneal only and 28.3% (13/46) of pelvic and retroperitoneal regions. Overall 52 symptoms representing toxicities were observed before ART, 107 during ART, 88 after end of ART and 52 at latest follow up. Leading toxicities during ART were diarrhoea (19%, 20/107), urinary incontinence (16%, 17/107) and fatigue (16%, 17/107). The spectrum of late toxicities was almost equal to those before beginning of ART. No grade 3 adverse events or chronic lymphedema at extremities were observed. We observed no clear correlation between localisation of treated regions, technique of ART and frequency or severity of toxicities. Mean quality of life at final evaluation was 74%.

**Conclusion:**

ART after extended LND in PCa relapse is justifiable with respect to adverse effects and toxicity. The side effects were circumscribed and well tolerated. The spectrum of adverse events at latest follow up was almost equal to those before start of ART.

## Introduction

Radical prostatectomy (RP) and radiotherapy (RT) are the standard treatment options for clinically localized prostate cancer (PCa) [1]. However relapses after primary treatment of PCa occurs depended on initial tumor stages from 10 to 53% [2]. Different recurrence patterns exist: (1) evidence of only local recurrence in the prostatectomy bed; (2) evidence of loco-regional metastases in pelvic lymph nodes (3) distant metastases (nodal, soft tissue, osseous) and (4) a combination of local and distant metastases [3]. Salvage RT is the mainstay therapy in the setting of local recurrence in the prostatic fossa and it offers the potential of cure [4–6].

Magnetic Resonance Imaging (MRI) and Positron Emmission Tomography/Computed Tomography (PET/CT) using 18 F- or 11C-choline have the potential to accurately identify the site of recurrence [5, 7]. Choline-PET/CT showed high region based sensitivity and specificity for detection of lymph node recurrence [8]. Nodal recurrent PCa after primary treatment or after salvage RT of the prostate fossa is considered an unfavourable situation and androgen deprivation is administered as standard therapy in this tumor stage. Antihormonal therapy causes serious side effects and is of limited benefit due to development of castration resistant PCa and therefore serves merely as palliative therapy [9, 10].

However there is increasing evidence that local ablative therapy of LNM in the primary situation could reduce the risk of progression [11] and it has been discussed, that there may be a different outcome between patients with solitary or few pelvic LNM compared to patients with bone metastases [12].

Recently it has been reported that choline-PET/CT guided salvage lymph node ablation therapy, either done by surgery, surgery followed by adjuvant radiotherapy (ART) or radiotherapy alone may be an effective strategy with long term disease control and possible curative potential [13–16]. Modern techniques such as image guided ART enable the treatment of involved regions after LND. This combination therapy is still investigational, but appears reasonable while detection rates of a single metastatic node by choline-PET/CT is limited by the spatial resolution of 5 mm [8] and removal rates of salvage surgery may be limited by altered lymphatic spread due to prior surgical intervention [11].

There may be severe concern over anticipated side effects of such a therapeutic approach. For example lymphedema following treatment for gynecological cancer has been reported to occur in up to 20% [17–19]. To our knowledge there are no published data about combined LND and ART of involved lymph node regions in patients with nodal recurrence. Therefore the aim of the present study was first to evaluate acute and late toxicity of this experimental approach and second to obtain detailed information about late term quality of life and different functional scores.

## Material and methods

### Patients

43 patients with prostate specific antigen (PSA)-recurrence (PSA >0.2 ng/ml after radical prostatectomy, PSA 2 ng/ml above the nadir after primary radiotherapy in 2 consecutive measurements) and a choline-PET/CT [^11^C-choline or ^18^ F-fluorethylcholine-PET/CT] positive for lymph node metastases were treated with pelvic and/or retroperitoneal salvage LND at Freiburg-University-Hospital from 2005–2013. All PET/CT scans were performed as a whole body imaging protocol with an integrated multislice PET/CT scanner using intravenous and oral contrast medium for enhanced CT to obtain full diagnostic quality. A lesion was defined as focal tracer accumulation greater than background activity with a corresponding lymph node in pelvic or retroperitoneal regions in the coregistrated CT.

Inclusion criteria were verification of biochemical recurrence, presence of choline-PET/CT positive lymph node metastases (regardless of number) without detectable bone or visceral metastases, Charlson-Comorbidity-index ≤2, age <80 years. Antihormonal therapy, if administered beforehand, had to be discontinued for at least 8 weeks. All patients underwent additional bone-scintigraphy to confirm exclusion of skeletal metastases. 46 ART were performed. 3 patients had 2 salvage lymph node dissections and 2 ART at different sites. Because of the experimental character of the surgical intervention (salvage lymph node dissection) and the adjuvant radiotherapy the patients had to sign informed consent. Furthermore, the patients gave signed written consent with respect to this retrospective analysis. The local review board reviewed and approved the study (No. 135/12_130160).

### Salvage lymph node dissection

According to choline-PET/CT findings pelvic (10 subregions: common iliac vessels, external iliac vessels, obturatoria vessels, internal iliac vessels, presacral region) or/and retroperitoneal (4 subregions: aortic bifurcation, aortal, caval, interaortocaval) LND was performed. Salvage-LND included complete removal of lymphatic and fatty tissue. The genitofemoral nerve formed the lateral border for pelvic lymphadenectomy. All LNDs were performed by the same surgeon (WSS).

### Adjuvant radiotherapy

Pelvic and retroperitoneal great vessels served as guidance to define clinical target volume (CTV). The cranial border of the retroperitoneal LN-region were the renal vessels, the inferior border the aortic bifurcation. The cranial border of a pelvic region (left/right) was the aortic bifurcation. The lower border of the pelvic radiation port were the top of the femoral heads. An approximately 8–10 mm margin around the vessels was drawn to define the CTV. Planning target volume (PTV) was 5–7 mm around the CTV. The following dose restrictions to reduce normal tissue complication probability were used as guidelines in radiotherapy planning: small bowel V45 Gy <250 ml, V50 Gy < 100 ml; rectum V50 Gy <50%, bladder V55 Gy < 50% [20, 21]. LN-regions were treated five times a week with 1.8 Gy/fraction up to a mean dose of 49.6 Gy (median 50.4 Gy, SD: 4.16 Gy, range 45–59.4 Gy) with 3D conformal irradiation or Intensity Modulated Radiation Therapy (IMRT). Due to dose constraints in adjacent normal tissues (small bowel, colon) shrinking field technique in terms of dose escalation >45 Gy to involved LN-regions was performed in 11/46 cases. If imaging suggested presence of local recurrence (12/43 patients), based on MRI and choline-PET/CT findings, prostate fossa received dose escalation (mean 69.2 Gy). Volumes of prior irradiation were excluded. Linear accelerators with 6 and 10 MV photons were used equipped with electronic portal imaging.

### Sources for evaluation of toxicity

To retrospectively evaluate the toxicity of ART, 31/43 patients were physically consulted in the out-patient-facility of the Departments of Urology or Radiation Oncology. A catalogue containing 15 selected items of possible adverse events/side effects (constipation, diarrhoea, rectal bleeding, nausea/vomiting, haematuria, urinary incontinence, dysuria (including: urgency, feeling of obstruction, pain), skin erythema in radiated region, skin hyperpigmentation in radiated region, lymphedema lower extremity, lymphocele, paraesthesia, fatigue/exhaustion, thrombosis, embolism) was extracted from the CTCAE-classification-list [22] and applied for every patient resp. every performed ART. Side effects were related to different time points (before ART, during ART, after ART and at latest follow up). Physical examination of the patients (abdomen, skin, lower extremity) and a comprehensive review of the medical records from the Departments of Urology and Radiation Oncology completed the assessment. 31/43 (72%) patients had been physically examined and consulted. 5/43 (11.5%) patients were out of the question of being invited due to death during follow up. Those 5 patients died because of progressive PCa, time from end of ART to death was 1.5, 3.5, 3.8, 5.9 and 2.7 years. 5/43 (11.5%) were missed because of extraordinary long journey from residence to Freiburg Hospital and 2/43 (5%) refused the invitation for an extra examination at latest follow up.

### EORTC questionnaires

Both, QLQ-C30 and PR25 questionnaires were developed by the European Organization for Research and Treatment of Cancer (EORTC) Quality of Life Study Group [23, 24]. The QLQ-C30 contains 30 items (physical, role, emotional, cognitive and social function and global health status). Furthermore, several specific physical symptoms such as fatigue, nausea/vomiting, pain, dyspnoea, sleep disturbance, loss of appetite, constipation, diarrhoea and financial difficulties are covered. Each item is scored from 1 to 4 (1 = “not at all”, 2 = “a little”, 3 = “quite a bit”, 4 = “very much”). Global quality is scored from 1 (very poor) to 7 (excellent). The PR25 questionnaire is a prostate specific module to be used in combination with the QLQ-C30 questionnaire and contains 25 items (urinary symptoms – 9 items, bowel symptoms – 4 items, treatment related symptoms – 6 items, sexual function – 6 items). A high score for a functional scale represents a high, healthy level of functioning; a high score for the global health status QoL represents a high quality of life. A high score for a symptom scale item represents a high level of symptomatology. Questionnaire data were processed according to the EORTC QLQ-C30 scoring manual [25].

Data from prostate cancer module PR25 were preceded according to the recommendations of the EORTC Quality of Life Study Group. Missing items were treated according to the recommendations given in the manual: if at least half of the items from the scale had been answered, it was assumed that the missing items had values equal to the average of those items which were present for that respondent [24].

### Statistical analysis

Descriptive statistic was done by calculating means, medians and standard deviations. Continuous variables were compared with a two-sided unpaired t-test. Chi-square-test was used for analyzing contingency tables. Significance was assumed if p < 0.05. Cronbach’s coefficient α was calculated to analyse the internal consistency of the scales from the questionnaires. All statistics were done with SPSSv19 (IBM Corp. Armonk, NY, USA).

## Results

**Figure 1:**
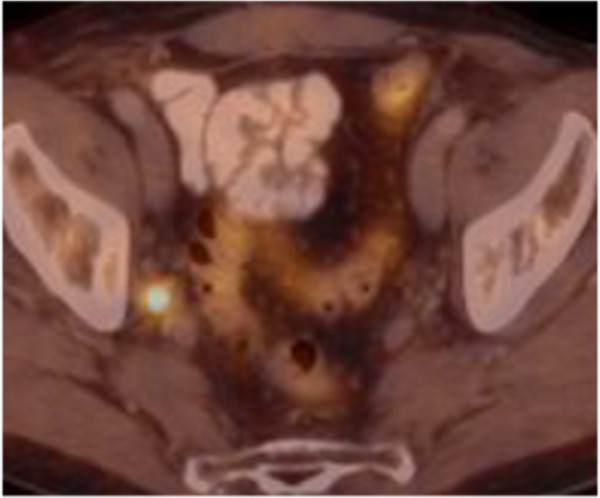
**Patient with two choline-PET/CT positive lymph nodes in the right obturator region in 09/12.** The second lymph node metastases was located at the iliaca communis subregion.

**Figure 2:**
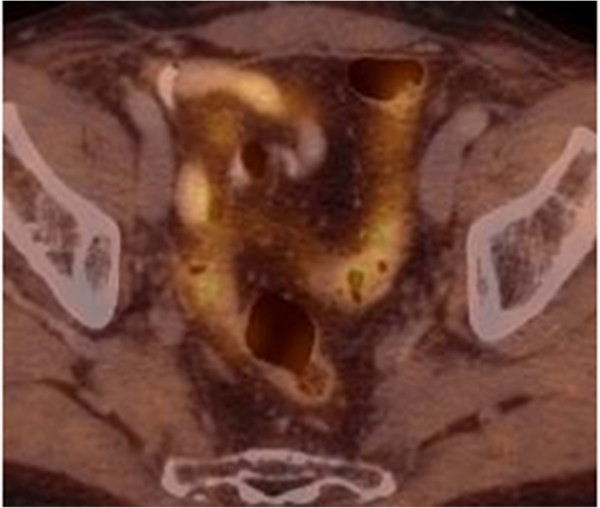
**Choline-PET/CT of the same patient as in Figure** 1 **in 10/13.** After salvage lymph node dissection and adjuvant radiation of the pelvic regions, there was no evidence for pelvic lymph node recurrence.

**Figure 3:**
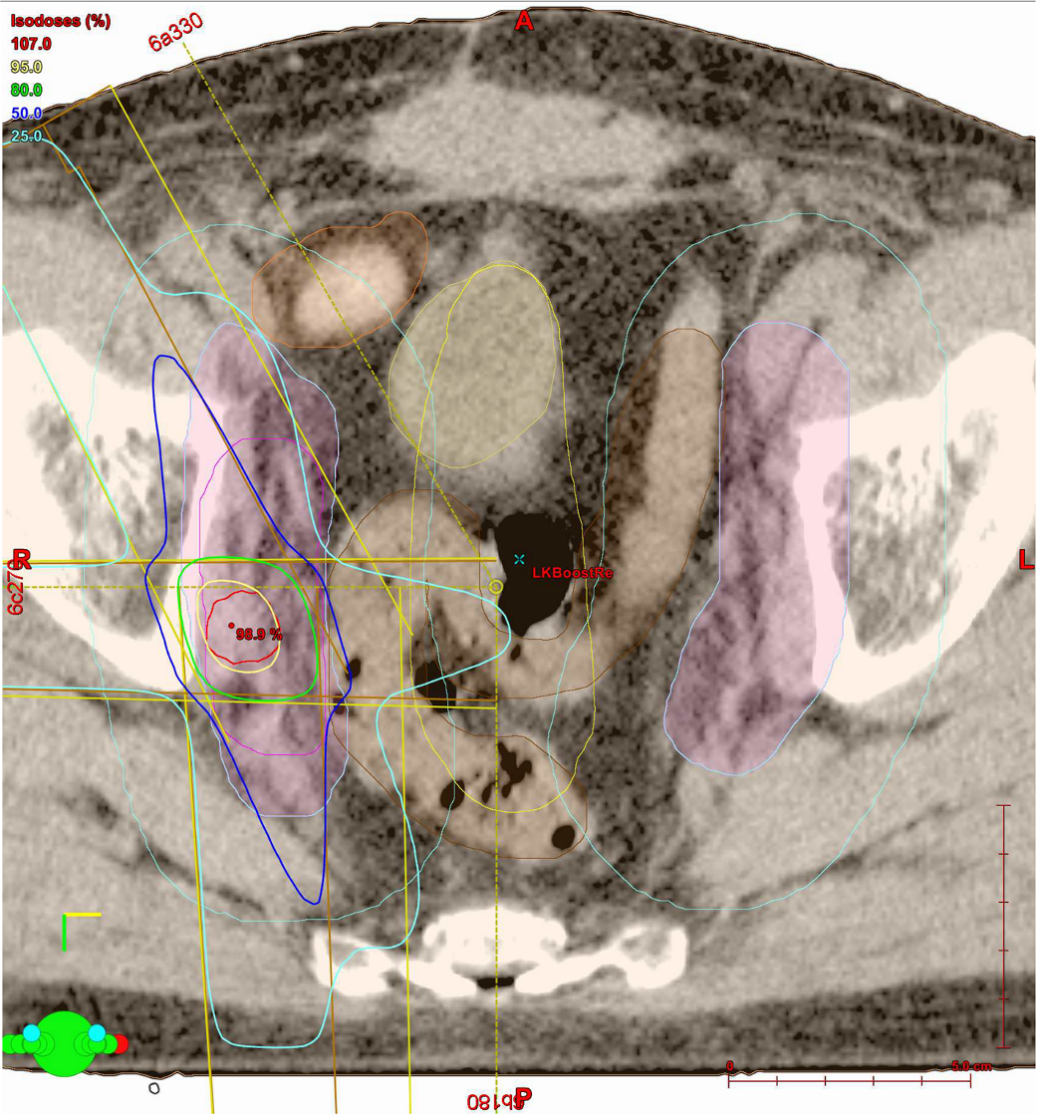
**Same patient as in Figures** 1 **and** 2 **.** Picture shows an example of the IMRT-Boost-plan that was influence by the choline-PET/CT-findings. This patient received local boost irradiation up to a sum dose 56,6 Gy.

Patient characteristics with respect to initial PCa stage and primary therapies from 43 individuals are shown in Table 1. Table 2 presents results from 46 LND performed in 43 patients because of nodal PCa relapse after primary therapy. All 43 patients underwent choline-PET/CT-imaging before lymphadenectomy. ART after LND was performed in 46 cases. Data from 46 ARTs with respect to region, total dose, the performance of a boost in affected regions and concurrent radiation of prostatic fossa are given in Table 3.Figure 1 shows a representative choline-PET/CT from a patient with positive lymph nodes in the right obturator region in 09/12, the second lymph node was located at the iliaca communis subregion. Figure 2 shows a choline-PET/CT of the same patient in 10/13 after LND and ART of the pelvic lymph node regions. There was no evidence for pelvic lymph node recurrence. The IMRT-Boost plan influenced by choline-PET/CT findings is shown in Figure 3. This patient received local boost irradiation up to a sum dose 56,6 Gy.

**Table 1:** **Patient characteristics regarding initial prostate cancer stage and primary therapy from 43 patients**

Variables	Value
Primary therapy	
Radical prostatectomy n (%)	38/43 (88.4%)
Radiotherapy n (%)	5/43 (11.6%)
Age at primary therapy (years)	
Mean/SD/median/range	60.3/6.1/60.0/74 - 46
Pathologic Gleason score at primary therapy n (%)	
6	4/43 (9%)
7	21/43 (49%)
8	7/43 (16%)
9	9/43 (21%)
x	2/43 (5%)
pT-stage at primary therapy n (%)	
T2	16/43 (37%)
T3	26/43 (61%)
T4	1/43 (2%)
pN-stage at primary therapy n (%)	
pN0	27/43 (63%)
pN1	12/43 (28%)
pNx	4/43 (9%)
Positive surgical margin at primary therapy n (%)	
R0	17/43 (39%)
R1	14/43 (33%)
Rx	12/43 (28%)
Initial PSA at primary therapy (ng/ml)	
Mean/SD/median/range	16.4/14.83/10.9/4.2 – 24.96

Salvage-LND = Salvage lymph node dissection; SD = Standard deviation.

PSA = Prostate specific antigen (ng/ml).

**Table 2:** **Data from 46 salvage lymph node dissections from 43 patients with nodal prostate cancer relapse**

Variables	Value
Age at salvage lymph node dissection (years)	
Mean/SD/median/range	64.9/5.9/65.2/75 - 53
Time from primary therapy to salvage lymph node dissection (years)	
Mean/SD/median/range	4.9/3.4/4.4/15.7 – 0.4
Regions affected with lymph node metastases (Histopathology) n (%)	
Pelvic only	31/46 (67%)
Retroperitoneal only	5/46 (11%)
Pelvic and retroperitoneal	9/46 (20%)
Retroclavicular and retroperitoneal	1/46 (2%)
Number of lymph nodes removed per surgery	
Mean/SD/median/range	29.3/14.6/29.5/2 - 62
Number of removed lymph nodes metastases per surgery	
Mean/SD/median/range	7.9/4.5/8.6/1 - 44
PSA at salvage lymph node dissection (ng/ml)	
Mean/SD/median/range	8.7/14.5/3.2/0.57 – 72.62

**Table 3:** **Data from 46 adjuvant radiotherapies from 43 patients**

Variables	Value
Age at adjuvant radiotherapy (years)	
Mean/SD/median/range	64.8/5.81/65.3/75 - 54
Time from salvage lymph node dissection to adjuvant RT (months)	
Mean/SD/median/range	2.75/1.72/2.20/8.4 - 0.8
Region for adjuvant RT n (%)	
Pelvic right only	2/46 (4.3%)
Pelvic left only	5/46 (10.9%)
Pelvic only (left/right/bilateral)	26/46 (57%)
Retroperitoneal only	5/46 (11%)
Pelvic and retroperitoneal	14/46 (30%)
Retroclavicular and retroperitoneal	1/46 (2%)
Cases with dose escalation on involved region n (%)	11/46 (24.0%)
Method of adjuvant radiotherapy	
3D-conformal radiotherapy n (%)	29/46 (63%)
IMRT n (%)	17/46 (37.0%)
Concurrent radiation of prostatic fossa n (%)	12/46 (26.1%)
Total dose for radiation of prostatic fossa (Gy)	
Mean/SD/median/range	69.18/2.38/70.20/45.0 – 72.2
Duration of adjuvant radiotherapy (months)	
Mean/SD/median/range	1.39/0.35/1.37/2.4 – 1.0
Dose per fraction (Gy)	1.8, 5x1.8/week
Total dose for pelvic or retroperitoneal RT (Gy)	
Mean/SD/median/range	49.55/4.16/50.40/45.0 – 59.4
Number of cases with concurrent AHT at radiotherapy n (%)	2/46 (4.3%)
Patients with concurrent AHT at latest follow up	27/43 (62.8%)
Comorbidities at latest follow^1^ up n (%)	
Cardiovascular disease n (%)	10/38 (26.4%)
Diabetes mellitus n (%)	3/38 (7.9%)
Concurrent malignant disease n (%)	3/38 (7.9%)
Chronic disease/chronic pain n (%)	6/38 (15.9%)

RT = Radiotherapy.

AHT = Antihormonal therapy.

IMRT = Intensity-modulated radiation therapy.

^1^Mean 3.2 (SD: 2.8) years after end of radiotherapy.

Time from end of ART to first follow-up visit was mean 2.3 months (median 1.95 months, SD: 1.19 months, range: 6.23 – 0.9 months). Time from end of ART to latest follow up was mean 3.2 years (median 2.7 years, SD: 2.89 years, range: 10.1 -0.2 years).

**Figure 4:**
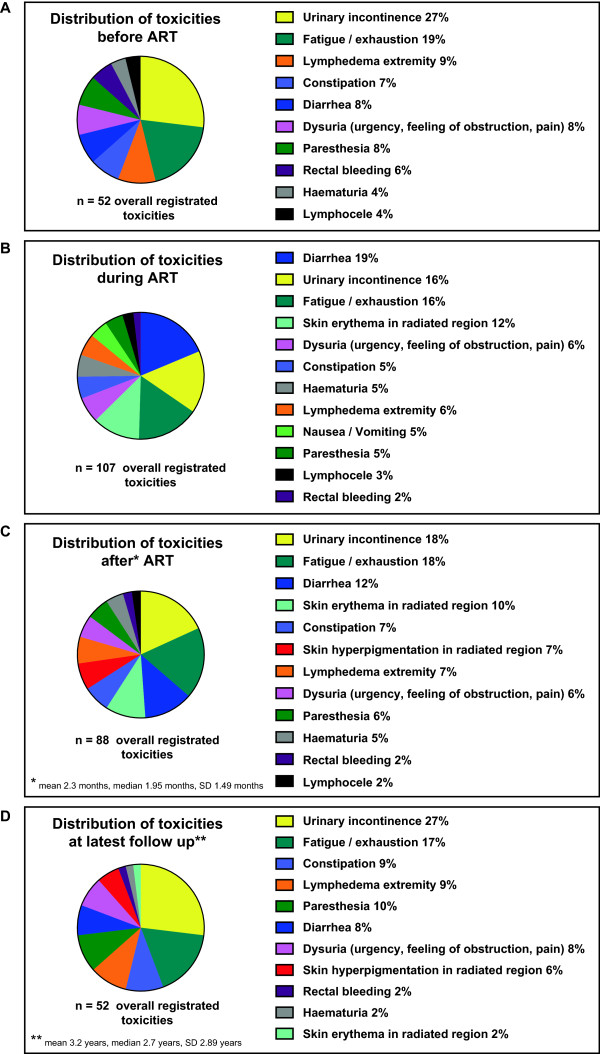
**Distribution (n, %) of overall recorded toxicities at different time points: A) before, B) during, C) after and D) at latest follow up from 46 radiotherapies.** The coloured panels reflect the percentage distribution of adverse events at different time points regardless of the CTCAE-grading.

Distribution of overall toxicities, regardless of the CTCAE-grading, before ART, during ART, after ART and at latest follow up, are shown in Figure 4A-D. Toxicity during ART (Figure 4B) and toxicity after ART (first follow up visit, Figure 4C) reflect the acute side effects and the therapy related symptoms at latest follow up reflect late toxicity. Before beginning of ART 52 therapy related ‘baseline’ symptoms were recorded resp. 107 symptoms during ART, and 88 after end of ART (first follow up visit), 52 therapy related symptoms were recorded at latest follow up (Figure 4A-D). Proportion of patients affected from one symptom of 15 recorded items at the different time points are shown in Table 4.

**Table 4:** **Evaluation of frequent side effect before/during/after ART and at timepoint of evaluation in 43 patients from 46 adjuvant radiotherapies**

Variables	Value	CTCAE ^3^-classification
**Constipation**		
Before ART	4/46 (8.7%)	4/4 (100%) grade 1
During ART	6/46 (13.0%)	6/6 (100%) grade 1
After ART^1^	6/46 (13.0%)	6/6 (100%) grade 1
Latest follow up^2^	5/37 (13.5%)	5/5 (100%) grade 1
**Diarrhea**		
Before ART	4/46 (8.7%)	4/4 (100%) grade 1
During ART	20/46 (43.5%)	15/20 (75.0%) grade 1/5/20 (25.0%) grade 2
After ART^1^	11/46 (23.9%)	11/11 (100%) grade 1
Latest follow up^2^	4/37 (10.8%)	4/4 (100%) grade 1
**Rectal bleeding**		
Before ART	3/46 (6.5%)	3/3 (100%) grade 1
During ART	2/46 (4.3%)	2/2 (100%) grade 1
After ART^1^	2/46 (4.3%)	2/2 (100%) grade 1
Latest follow up^2^	1/37 (2.7%)	1/1 (100%) grade 1
**Nausea/Vomiting**		
Before ART	0/46 (0.0%)	-
During ART	4/46 (8.7%)	4/4 (100%) grade 1
After ART^1^	0/46 (0.0%)	-
Latest follow up^2^	0/37 (0.0%)	-
**Haematuria**		
Before ART	2/46 (4.3%)	2/2 (100%) grade 1
During ART	6/46 (13.0%)	6/6 (100%) grade 1
After ART^1^	4/46 (8.7%)	4/4 (100%) grade 1
Latest follow up^2^	1/37 (2.7%)	1/1 (100%) grade 1
**Urinary incontinence**		
Before ART	14/46 (30.4%)	12/14 (85.7%) grade 1/2/14 (14.3%) grade 2
During ART	17/46 (36.9%)	8/17 (47.1%) grade 1/9/17 (52.9%) grade 2
After ART^1^	16/46 (34.7%)	9/16 (56.3%) grade 1/7/16 (43.7%) grade 2
Latest follow up^2^	14/37 (37.8%)	10/14 (71.4%) grade 1/4/14 (28.6%) grade 2
**Dysuria (urgency, feeling of obstruction, pain)**		
Before ART	4/46 (8.7%)	4/4 (100%) grade 1
During ART	7/46 (15.2%)	7/7 (100%) grade 1
After ART^1^	5/46 (10.9%)	5/5 (100%) grade 1
Latest follow up^2^	4/37 (10.8%)	4/4 (100%) grade 1
**Skin Erythema in radiated region**		
Before ART	0/46 (0.0.%)	-
During ART	13/46 (28.3%)	13/13 (100%) grade 1
After ART^1^	9/46 (19.6%)	6/9 (66.7%) grade 1/3/9 (33.3%) grade 2
Latest follow up^2^	1/37 (2.7%)	1/1 (100%) grade 1
**Skin hyperpigmentation in radiated region**		
Before ART	0/46 (0.0.%)	-
During ART	0/46 (0.0.%)	-
After ART^1^	6/46 (13.0%)	6/6 (100%) grad 1
Latest follow up^2^	3/37 (8.1%)	3/3 (100%) grade 1
**Lymphedema extremity**		
Before ART	5/46 (10.9%)	5/5 (100%) grade 1
During ART	6/46 (13.0%)	6/6 (100%) grade 1
After ART^1^	6/46 (13.0%)	6/6 (100%) grade 1
Latest follow up^2^	5/37 (13.5%)	5/5 (100%) grade 1
**Lymphocele**		
Before ART	2/46 (4.3%)	2/2 (100%) grade 1
During ART	3/46 (6.5%)	2/3 (66.7%) grade1/1/3 (33.3%) grade 2
After ART^1^	2/46 (4.3%)	2/2 (100%) grade 1
Latest follow up^2^	0/37 (0.0.%)	-
**Paresthesia**		
Before ART	4/46 (8.7%)	4/4 (100%) grade 1
During ART	5/46 (10.9%)	5/5 (100%) grade 1
After ART^1^	5/46 (10.9%)	5/5 (100%) grade 1
Latest follow up^2^	5/37 (13.5%)	5/5 (100%) grade 1
**Fatigue/exhaustion**		
Before ART	10/46 (21.7%)	10/10 (100%)
During ART	17/46 (37.0%)	17/17 (100%)
After ART^1^	16/46 (34.8%)	16/16 (100%)
Latest follow up^2^	9/37 (24.3%)	9/9 (100%)
**Thrombosis**		
Before ART	0/46 (0.0.%)	-
During ART	0/46 (0.0.%)	-
After ART^1^	0/46 (0.0.%)	-
Latest follow up^2^	0/37 (0.0.%)	-
**Embolism**		
Before ART	0/46 (0.0.%)	-
During ART	0/46 (0.0.%)	-
After ART^1^	0/46 (0.0.%)	-
Latest follow up^2^	0/37 (0.0.%)	-

ART = adjuvant radiotherapy.

^1^Mean 2.3 (SD: 1.2) months after end of radiotherapy.

^2^Mean 3.2 (SD: 2.8) years after end of radiotherapy.

^3^Common Terminology Criteria for Adverse Events Version 4.0.

Only with respect to urinary incontinence, diarrhoea, skin erythema and development of lymphoceles grade 2 toxicities had been observed in our study. Grade 2 urinary incontinence during ART was observed in overall 9 cases, 3 of those 9 adverse events occurred upon pelvic and retroperitoneal ART, 5/9 events occurred upon pelvic only ART, one case of urinary incontinence was associated with retroperitoneal only ART. 5 cases with grade 2 diarrhoeas were observed. 3 of those 5 occurred from ART of pelvic and retroperitoneal lymph node regions, 2/5 grade 2 diarrhoeas occurred from pelvic only ART. 6/9 patients who complained about grade 2 urinary incontinence had been treated with 3D-conformal radiation technique; the remaining 3/9 individuals had been treated using IMRT. 4/5 patients with grade 2 diarrhoea were treated with 3D-ART, the remaining individual was treated with IMRT.

By evaluating 15 symptoms/items (Table 4) during radiotherapy from 17 IMRTs and 29 3D-ARTs, the overall rate of toxicity, regardless of the grading, was compared between both techniques. In the IMRT-group 44 of 255 possible toxicities were observed, 63 of 435 possible toxicities were recorded in the 3D-ART group. There was no significant difference between both groups (p-value: 0.33).

Diarrhoea and urinary incontinence were the leading symptoms during ART (Figure 4B and Table 4). 8 events of diarrhoea during ART were recorded upon 14 pelvic and retroperitoneal treatments resp. 12 cases of diarrhoea occurred upon 26 ARTs of the pelvis only. With respect to the extent of the irradiated regions the frequency of diarrhoea (regardless of grading) was not significant different between both groups (p-value: 0.74).

Upon 26 pelvic ARTs, 8 events of urinary incontinence during ART were recorded. Upon 14 pelvic and retroperitoneal ARTs 4 cases of urinary incontinence were noticed. No significant difference was observed between both conditions (p-value: 0.33).

Generally the fraction of patients with urinary incontinence before start of ART (14/46) was not significant different (p-value: 0.494) from the fraction of patients with incontinence at latest follow up (14/37). Also the fraction of patients with diarrhoea before start of ART (4/46) was not significant different (p-value: 1.0) from the fraction of patients who had diarrhoea at latest follow up (4/37).

5 events with paraesthesia during ART were recorded (Table 4). 4/5 paraesthesias had already been noticed before ART (1/5 general polyneuropathy, 1/5 right inguinal, 1/5 inner region thigh, 1/5 inguinal right). No progression during or after ART was observed. Only one patient (1/5) complained about newly diagnosed paraesthesia (inguinal left and right and thigh right) that had not existed before ART.

2 patients had residual lymphoceles from salvage LND, without indication for drainage because of small size and lacking symptoms (Table 4). One patient developed a pelvic lymphocele during ART with indication for drainage, clearly related to performed salvage lymphadenectomy recently before.

6 patients showed lymphedema (grade 1) during ART. 5/6 of those had lymphedemas already before beginning of ART (2/6 swelling upper and lower leg right post salvage LND, 1/6 ankle oedema, 1/6 lower leg oedema left, 1/6 upper leg oedema with varicosis). 1 patient developed newly grade 1 lymphedema at the right upper leg region.

Hematopoetic parameters (level of haemoglobin, leukocytes, thrombocytes) at different time points are shown in Table 5. A significant difference before and during ART was observed only for the level of leucocytes (6,600/μl versus 4,800/μl, p-value 0.0009, CI: 2.75 - 0.75).

**Table 5:** **Hematopoetic parameters before, during and after adjuvant radiotherapy in 46 cases from 43 patients**

Hemoglobin	Gram/dl
Before ART^1^	13.8/1.79/14.0 (mean/± SD/median)
During ART^2^	13.01/1.37/13.0 (mean/± SD/median)
After ART^3^	13.34/2.03/13.85 (mean/± SD/median)
Cases with Hemoglobin-decline during ART	3/46 (6.5%)
**Leukocytes**	**Thousand/μl**
Before ART^1^	6.58/2.07/6.30 (mean/± SD/median)
During ART^2^	4.81/1.53/4.40 (mean/± SD/median)
After ART^3^	5.92/2.08/5.07 (mean/± SD/median)
Cases with Leukocytes-decline during ART	8/46 (17.4%)
**Thrombocytes**	**Thousand/μl**
Before ART^1^	240.43/74.84/226.50 (mean/± SD/median)
During ART^2^	204.17/71.84/192.0 (mean/± SD/median)
After ART^3^	228.26/64.03/231.0 (mean/± SD/median)
Cases with Thrombocytes-decline during ART	2/46 (4.5%)

ART = adjuvant radiotherapy.

^1^Mean 2.4 months before start of ART (SD: 2.5 months, median 1.78 months).

^2^Mean 0.80 months after start of ART (SD: 0.46 months, median 0.70 months).

^3^Mean 2.9 months after end of ART (SD: 3.15 months, median 1.57).

At latest follow up 21/43 of the patients (46%) were ECOG 0 status, 14/43 (30%) were ECOG 1 status (not evaluable 24% (11/43)).

**Figure 5:**
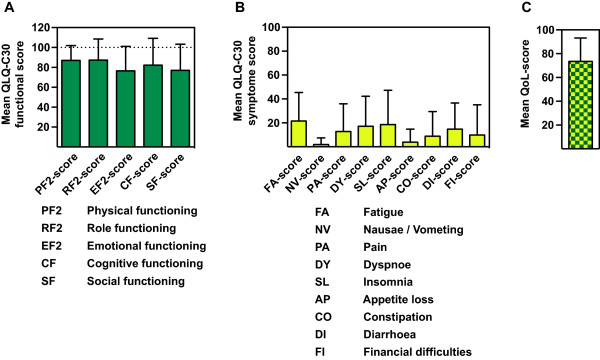
**Mean functional and symptoms scales and quality of life-score from the QLQ-C30 questionnaires.** Mean functional scales **(A)**, symptoms scales **(B)** and **(C)** quality of life-score from the QLQ-C30 questionnaires analysed from (31/43, 72.1%) patients evaluated mean 3.2 years after end of radiotherapy.

**Figure 6:**
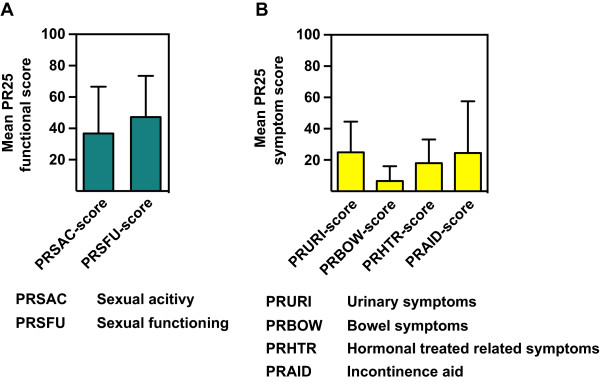
**Mean functional and symptoms scales from the prostate cancer module QLQ-PR25 questionnaires.** Mean functional scales **(A)** and symptoms scales **(B)** from the prostate cancer module QLQ-PR25 questionnaires analysed from (31/43, 72.1%) patients evaluated mean 3.2 years after end of radiotherapy.

Results of EORTC quality of life (QLQ-C30) and EORTC prostate cancer module (QLQ-PR25) from 36 patients that had been physically consulted at latest follow up are shown in Tables 6 and 7 resp. in Figures 5 and 6. Calculated Cronbach’s alpha for PRURI-score, PRBOW-score, PRHTR-score, PRSAC-score and PRSFU-score was 0.82, 0.34, 0.50, 0.64 and 0.53 respectively. Calculated Cronbach’s alpha for PF2-score, RF2-score, EF2-score, CF-score, SF-score, FA-score, NV-score, PA-score and QL2-score was 0.64, 0.91, 0.90, 0.74, 0.81, 0.84, 0.73, 0.76 and 0.88 respectively. These data indicate a good to excellent internal consistency in most of the checked items.

**Table 6:** **EORTC quality of life questionnaire (QLQ-C30) at timepoint of evaluation [Mean 3.2 (SD: 2.8) years after end of radiotherapy]**

QLQ-C30 functional scores	Value (mean/± SD/median)
Physical functioning (PF2 –score)	87.1/14.9/93.3
Role functioning (RF2-score)	87.3/21.3/100.0
Emotional functioning (EF-score)	76.5/24.6/91.7
Cognitive functioning (CF-score)	82.8/27.2/100.0
Social functioning (SF-score)	77.0/26.6/83.3
**QLQ-C30 symptom scores**	**Value (mean/± SD/median)**
Fatigue (FA-score)	21.6/23.8/16.7
Nausea/Vomiting (NV-score)	2.0/5.5/0.0
Pain (PA-score)	12.7/25.4/0.0
Dyspnea (DY-score)	17.2/25.2/0.0
Insomnia (SL-score)	18.6/28.7/0.0
Appetite loss (AP-score)	3.9/10.9/0.0
Constipation (CP-score)	8.8/20.9/0.0
Diarrhea (DI-score)	14.7/22.0/0.0
Financial difficulties (FI-score)	9.8/25.3/0.0
**Quality of life**	**Value (mean/± SD/median)**
QoL-score	74.0/19.7 / 83.3

**Table 7:** **EORTC prostate cancer module (QLQ-PR25) at timepoint of evaluation [Mean 3.2 (SD: 2.8) years after end of radiotherapy]**

PR25 functional scores	Value (mean/± SD/median)
Sexual activity (PRSAC-score)	36.8/29.4/33.3
Sexual functioning (PRSFU-score)	47.2/25.4/33.3
**PR25 Symptom scores**	**Value (mean/± SD/median)**
Urinary symptoms (PRURI-score)	24.9/20.8/19.5
Bowel symptoms (PRBOWE-score)	6.6/9.2/0.0
Hormonal treatment-related symptoms (PRHTR-score)	18.0/14.9/16.7
Incontinence aid (PRAID-score)	24.6/32.2/0.0

## Discussion

The results of this study showed that salvage ART after extended LND in prostate cancer relapse has acceptable low acute and late toxicity resulting in a high quality of life at mean 3.2 years afterwards. 88.4% of all patients had lymphadenectomy at primary therapy when radical prostatectomy and lymphadenectomy was performed (Table 1). However the second salvage lymphadenectomy was performed as an extended surgery represented by the mean number of 29.3 lymph nodes (Table 2). With additional adjuvant irradiation the risk of lymphedema may be considered fairly high due to at least threefold treated and possibly altered pelvic or retroperitoneal lymph node regions but in fact was not. Similarly in contrast to reported toxicities of adjuvant irradiation in gynaecological cancer a recent published series of patients that underwent pelvic irradiation after extended pelvic lymphadenectomy because of node-positive PCa in the primary situation showed low incidence of lymphedema with only mild characteristic [26]. In this series 26 patients underwent combined treatment for high-risk node-positive prostate cancer consisting of extended pelvic lymphadenectomy followed by androgen deprivation therapy and radiotherapy of pelvic lymph nodes, prostate including seminal vesicles with external beam radiotherapy (50 Gy) followed by brachytherapy boost of 2×10 Gy to the prostate only. With a median follow up of 2.2 years six patients (27%) experienced grade 1 lymphedema and two patients (9%) grade 2 while none had grade 3 or 4 according to the CTC Common Toxicity Criteria scale 4.0 including only mild urinary and rectal side effects [26]. Another series of 39 patients that received adjuvant pelvic IMRT because of node-positive prostate cancer in the primary situation has been published by Müller et al. [27]. Pelvic IMRT to 45–50.4 Gy was applied either after previous surgery including lymphadenectomy (n = 18) or with a radiation boost to suspicious nodes (n = 21) with doses of 60–70 Gy. They reported no lymphedema and only moderate acute radiation-related genitourinary and gastrointestinal toxicity (Grade 1–2), while 2 patients had potential severe toxicities of G3-4 (with the need for urinary catheter/subileus related to adhesions after surgery). Late toxicity was mild (Grade 1–2) after a median follow up of 70 months/5.8 years and over 50% of the patients reported no late morbidity. In their study in the group with lymphadenectomy a mean of 13 nodes had been removed. These and our studies are in contrast to data from gynaecologic studies that investigated toxicity after surgery and adjuvant radiation in cervical cancer: Landoni et al. reported severe edema of the legs in 9% who had surgery plus adjuvant radiotherapy [28]. A recent retrospective study on the prevalence of lymphedema after gynecological cancer treatment (n = 802) showed prevalence of lymphedema in 10% and presence of symptomatic lower limb swelling in further 15%. However diagnosed lymphedema was more prevalent (36%) amongst vulvar cancer survivors and cervical cancer survivors who had radiotherapy or lymph node dissection had higher odds of developing swelling [17]. A retrospective study on the prevalence and incidence of lower limb lymphedema following treatment for gynaecological cancer (n = 487) showed that 36% of the women reported swelling of their legs with clincically diagnosed lymphedema in 18% [19]. Patients were most likely to develop lymphedema if they underwent adjuvant radiotherapy after dissection of lymph nodes in the groin region. No groin lymph node dissection was performed in our series, because the typically involved lymph nodes in prostate cancer patients with nodal recurrence are the proximal iliaca external, internal and iliaca communis subregions [8]. Additionally we investigated the possible effect of IMRT vs. 3D conformal radiation technique on the prevalence of side effects, but there was no significant difference between both techniques. Only 24% received dose escalation of the involved lymph node subregion, while in these cases the total dose did not exceed 59.4 Gy. The reason for dose escalation was the presence of histological confirmed extensive lymphatic spread in a lymph node subregion with a higher risk of residual disease despite salvage lymph node dissection. However also in such situations normal tissue dose restrictions as mentioned in the methods section were strictly followed, explaining the low toxicities. Mild Diarrhoea and mild urinary incontinence were frequent in the spectrum of toxicities. Both are typical acute side effects of pelvic/retroperitoneal irradiation [26, 27]. However long term toxicity was lower than acute side effects and the spectrum of late toxicity matched the spectrum before adjuvant radiotherapy indicating nearly complete healing up of side effects (Figure 4). Fatigue was the second most symptom before and after ART but did not increased at latest follow up. Our data showed are considerably lower prevalence and severity of long term side effects than a recent study on the effects of pelvic radiotherapy on cancer survivors including men and women (n = 418) [29]. Adams et al. described a higher incidence of moderate (grade 2) and severe (grade 3/4 problems with bowel, urinary and sexual functioning: bowel urgency (59% women, 45% men); urine urgency (49% women, 46% men); urine incontinence (38% women, 9% men). Study symptoms were just as frequent in those 6–11 years after treatment as in those 1–5 years after treatment. Symptom severity was significantly associated with poorer overall quality of life and higher levels of depression. The authors concluded that late effects are common among long-term cancer survivors who have had pelvic radiotherapy, and are associated with reduced quality of life. In contrast quality of life functional and symptom scores and global health status were somewhat similar to our results (mean 72% [29] vs. mean 74% (Table 6)). The higher gastrointestinal and urinary toxicity in their study compared to our data is on the one hand likely due to the inclusion of men with bladder and rectal cancer beside patients with prostate cancer, each entity requiring different target volumes. On the other hand no detailed information about radiotherapy and surgery has been reported in their study. In addition it is unclear which dose restrictions and volumes of irradiation have been used in their population. These considerations are likely to explain the difference to the mild grade of toxicity in our cohort.

A significant proportion of the patients reported presence of pain reflected by the PA-score of 12.7% (Table 6). The site of the pain in our cohort was not otherwise specified exept recording of dysuria. No gastrointestinal pain was documented in the patients health records and was not recalled at the final assessment. Other disease processes and comorbidities associated with ageing, such as musculo-skelettal disease also contribute to the general measurement of life quality. In our cohort pain was predominantly caused by degenerative musculo-skelettal diseases.

The fact that 62.8% of the patients were under antihormonal therapy mean 3.2 years after adjuvant radiotherapy indicates that the majority of patients had progressive disease despite an intensive local ablative concept. However recent data showed that a significant proportion of patients may profit by having long term disease control or delayed need of antihormonal therapy [13–16]. Further studies are needed to evaluate which subgroup of patients are likely to profit from this approach but this issue is beyond the scope of this study.

Our study has some limitations. First it is a retrospective study and some symptoms may have not been documented in the patients records before, during ART and at first follow up visit. However all patients received a weekly consultation during ART which is a standard procedure in the department of Radiation Oncology and it is not likely that significant side effects have not been recorded. To overcome this drawback all patients that followed the invitation for physical consultation were asked if they recall any side effects before, during and after ART. So if they stated a symptom that was not recorded in the original reports this recalled side effect was put to our statistics. Second it is a single center study of an experimental approach. Although valid guidelines for pelvic radiotherapy have been given and served as reference in the radiotherapy planning process [20, 21] salvage lymph node dissection is not a standard procedure and number of removed lymph nodes as well as postoperative morbidity depends on the experience of the surgery/urology department and the skills of the surgeon. Therefore our observed low toxicities despite prior lymph node dissection and second salvage lymph node dissection may be also the result of thorough experience with this procedure.

## Conclusion

Data of the present study indicate that extended salvage lymph node dissection followed by adjuvant radiotherapy is associated with low risk of only mild lymphedema even after previous lymph node dissection in the primary situation. Furthermore the characteristic of spectrum of therapy-associated side effects was mild before, during and after adjuvant radiotherapy and this combined approach may be regarded as a safe therapy. The spectrum of adverse events mean 3.2 years after end of ART was almost equal to those before start of ART. Choline PET-CT directed LND with adjuvant ART my serve as valid salvage concept with good clinical response and low side effects.
